# Hepatitis B Viral Infection and its Associated Factors among Population Aged at Least 15 Years in Three Selected Cities of Burundi

**DOI:** 10.24248/eahrj.v8i3.802

**Published:** 2025-01-30

**Authors:** Armstrong Ndihokubwayo, Tharcisse Nahimana, Emmanuel Hakizimana, Edouard Nkunzimana, Emile Ntirampeba, Cassien Nduwimana, Emmanuel Kayagwa, Nehemie Nzoyikorera, Joseph Nyandwi

**Affiliations:** a Institut National de Santé Publique, Laboratoire National de Référence, Burundi; b University of Ngozi, Faculty of Medicine and Health Sciences, Burundi; c Hope Africa University, Van Norman Clinic & Hope Africa University Hospital, Burundi; d Africa Centres for Disease Control and Prevention, Emergency Preparedness and Response Division, Addis Ababa, Ethiopia; e Kira Hospital, Department of Clinical Biology Laboratory, Burundi; f Mohamed VI University of Health Sciences (UM6SS), Higher Institute of Biosciences and Biotechnology, Casablanca, Morocco; g Mohamed VI University of Health Sciences (UM6SS), Mohamed VI Centre for Research & Innovation, Rabat; Laboratory of Microbial Biotechnology and Infectiology Research; h Université du Burundi, Faculté de Médecine; i Ministry of Public Health and the Fight against AIDS, National Institute of Public Health, Burundi; j Université Hassan II de Casablanca, Faculté de Médecine et Pharmacie, Laboratoire d’Immologie clinique, Inflammation et Allergie.

## Abstract

**Background:**

Hepatitis B virus infection is a common cause of viral hepatitis and affects 257 million people worldwide. Hepatitis B virus disease is a potentially life-threatening liver infection and a major global health problem that puts people at high risk of death from cirrhosis and hepatocarcinoma. The present study sought to investigate the proportion of hepatitis B virus (HBV) and associated factors for its transmission among people aged at least 15 years in three selected cities of Burundi attending the HBV screening campaign.

**Methods:**

We conducted a cross-sectional design by selecting conveniently 629 consenting participants aged at least 15 years during the screening campaign in three selected cities of Burundi namely Gitega, Rumonge and Cankuzo in June 2022. During the campaign, a structured questionnaire was administered by trained healthcare workers to collect socio-demographic and behavioural characteristics, as well as the history of exposure to HBV. HBV screening tests were performed with Cypress Diagnostics HBsAg Cards (Hulshout, Belgium). Univariate and multivariable logistic regression analyses were used to assess factors associated with HBV infection in the screened participants.

**Results:**

The study included 629 participants, 8.43% of whom tested positive for hepatitis B viral infection, with a mean age of 37.84 (SD=13.9) years. The participants were predominantly found in the over 50 years age group (31.1%) and the 18–30 years age group at 26.69%, the married (70.6%), the female (57.71%), and the farmers (60.25%), Rumonge city dwellers (33.39%), and those with a primary school level (36.25%). In this study, the associated factors with viral hepatitis B infection were residence in Cankuzo (OR=2, CI= 1–4, *p=.04*), and history of sharing sharp materials (OR=1.8, CI=1-3.3, *P=.03*).

**Conclusion:**

HBV infection was significantly associated with residence in Cankuzo and sharing sharp materials. HBV infection is endemic in these three provincial cities namely Cankuzo, Gitega and Rumonge. Given the various ways for HBV infection to occur within a general population, control of hepatitis B and its associated factors is one of the highest priorities in order to mitigate its transmission and monitor continuous exposure among Burundian population. There needs to be more help in the form of screening, immunizations for adults and other preventative measures, as well as treatment for the patients.

## BACKGROUND

Viral hepatitis is an international public health challenge, comparable to other major communicable diseases, including HIV, tuberculosis and malaria.^1^ Hepatitis B virus (HBV) infection is a common cause of viral hepatitis, and it affects 257 million people worldwide^2,3^. The HBV disease is a potentially life-threatening liver infection and a major global health problem which puts people at high risk of death from cirrhosis and hepatocarcinoma.^4–6^

The estimated overall seroprevalence of HBV surface antigen in Africa (6.1 %) and Western Pacific Region (6.2 %), remains high despite the introduction of universal HBV vaccination and effective antiviral therapy.^7^ Almost 8% of this global burden is in Sub-Saharan Africa, with over 80,000 new infections occurring each year.^3,8^ The WHO estimates that 70–95 % of the adult population in sub-Saharan Africa is exposed to different types of Viral Hepatitis (A, B, C, D, E) with Hepatitis B Surface Antigen (HBsAg) seroprevalence rates of 6 to 20 %.^9^

In East African countries, only a few studies have been conducted on the epidemiology of viral hepatitis in the general population, and more studies are fragmented and focused on specific subpopulations.^10–12^ In the neighbouring countries of Burundi (Democratic Republic of Congo, Rwanda, and Tanzania), the seroprevalence of hepatitis B infection ranges from 2 % to 5 % in different studies and in the general population.^10–12^ In Burundi, the current prevalence of the virus of hepatitis B is not well-known in the general population; but according to existing studies, the prevalence of HBV was estimated at 4.6% in 2002,^13^ and at 3.33% in blood donors in 2019.^14^

The modes of transmission of HBV vary in different geographical areas, the main routes of transmission are sexual intercourse, parenteral contact, and vertical transmission and contact with blood or derivatives during medical procedures.^14,15^ Hepatitis B Virus has three different antigens, HBsAg, HBeAg and HBcAg, and hepatitis B surface antigen (HBsAg) is used to diagnose and monitor the progress of Hepatitis B viral infection.^16^ A diagnosis of HBV infection requires laboratory confirmation by detection of HBsAg, a marker of active HBV infection.^17^ Screening for HBsAg is performed by immunochromatographic tests in different studies.^18^

Although the epidemiological situation of hepatitis B virus in Burundi is not well known, limited studies have been conducted on its seroprevalence. The limited information about its seroprevalence and associated factors among the general population is an obstacle to formulating effective policies and monitoring continuous exposure to reduce the burden of viral hepatitis. The objective of this study was to investigate frequency of HBV infection and associated factors for its transmission among Burundian population aged 15 years old and above during a screening campaign carried out in June 2022 in three selected cities of Burundi (Gitega, Rumonge and Cankuzo). Findings from this study would help decision makers understand the bottleneck with regard to Hepatitis B Virus and its associated factors and therefore to institute awareness programme to improve diagnosis, prevention, control measures, as well as therapeutic management.

## METHODS

### Study Design, Area and Population

The study was a cross-sectional study conducted in three selected provincial cities of Burundi namely Gitega (Centre), Rumonge (South-West) and Cankuzo (East) where the association of medical laboratory professionals conducted a viral hepatitis B screening campaign during the month of June 2022. The Three cities of Burundi were chosen based on their location, whereby Rumonge (South-West) and Cankuzo (East) are cities on the border with neighbouring countries and have many returning refugees and other migrants. This population could have a higher risk of HBV acquisition due to unhealthy living conditions, or possibly multicultural behaviours. Gitega (Centre) is a political capital city where the population is also multicultural; and could receive different visitors and tourists from different regions and countries. During the campaign, a structured questionnaire was administered by well-trained healthcare workers. The study population was constituted by any consenting individuals aged of at least 15 years attending the screening campaign. Sensitisation on HBV screening was done through multimedia announcements, local church leaders and community healthcare workers.

### Sample Size Determination and Sampling Technique

The sample size was calculated using a single proportion population formula based on the following assumptions: the assumption Z distribution with a 95% confidence interval (CI) was 1.96, the margin of error (d) was 3%, and proportion (p) was 5.2% as expected prevalence,^19^ with Q=1-P. The final sample size was computed using the Fisher’s formula:


$$n=\frac{Z_{\left(1-\alpha \right)}^{2}\text{PQ}}{d^{2}}$$

The study respondents included any consenting participants aged 15 years and above attending the HBV screening campaign; and were conveniently selected until the required sample size (629 participants) was achieved. This study excluded any participant who refused to provide blood for the study or to respond to the questionnaire. In total, 629 individuals who attended the HBV screening campaign and met the inclusion criteria were screened after verbal or signed consent was obtained.

### Independent and Dependent Variables

The primary outcome was a binary variable of viral hepatitis B infection, defined as “Yes” if participants tested HBsAg positive, and “No” if participants tested HBsAg negative. Independent variables were socio-demographic characteristics including age, sex, marital status, level of education, occupation, and city of residence. Behavioural variables were also assessed including history of VHB vaccination, HIV status, history of surgical operation, history of blood transfusion, history of family contact with HBV, history of multiple sexual exposure, exposure to a traditional operation practice and history of sharing sharps materials. The validity and reliability of dependent and independent variables questions developed by the authors were checked. The socio-demographic information and other behavioural details were self-reported by participants. Both Kirundi and French versions of a standardized questionnaire were used. Data collectors, with good communication skills and collection experience, were recruited from medical laboratory technologists. The supervision of the data collection process was done by collaborating authors on HBV screening sites.

### Laboratory Procedures

Under an aseptic technique, about (4 mL) of blood samples were obtained from all individuals, through venepuncture, using sterilized disposable 5 ml syringes and 20 gauge needles by laboratory technologists with good experience in the collection of biological samples. Cypress Diagnostics HBsAg Cards (Hulshout, Belgium) were used to test samples for hepatitis B surface antigen. The test card was removed from the kit and placed in the dry flat surfaces and 3 drops of buffer solution added by holding the pipette vertically and waiting for 15 to 20 minutes. Laboratory procedures and interpretation of results were done according to the manufacturer’s instructions (Notice of kits). HBV infection was defined as positive hepatitis B surface antigen. Laboratory results were mentioned on data collection forms.

For quality control, the appearance of red line in the control region (C) was the internal control and confirms correct procedure technique. The rapid immunochromatographic method used to detect HBsAg marker was proved by laboratory Standards in Burundi.

### Data Processing and Analysis

Filled forms were retrieved from data collectors, checked, validated, coded, entered into the computer using Microsoft Excel. Data were exported to STATA 12 software packages (version 12.0, College Station, TX) for analysis. The normality of continuous variables was analysed by the Shapiro–Wilk test. The distribution of quantitative variables was compared by the Student t-test for variables following normal distribution and the Man Whitney test for variables not following normal distribution. Qualitative variables were described as numbers, and proportions while quantitative variables as mean and standard deviation (SD).

Chi-square tests or Fisher’s exact test were used to measure the associations between categorical variables. In order to determine independent risk factors associated with viral hepatitis B among the screened participants, a multivariable logistic regression model with backward stepwise procedure included was performed. Variables included in the final multivariate model were those with a *p*-value ≤.25 in the univariate model. The level of significance for all the statistical analyses was set at 0.05.

### Ethical Consideration and Consent to Participate

Permission to conduct this study was sought from the Ministry of Public Health and Fight against AIDS and authorised by the offices of the participating health provinces (Rumonge, Gitega, and Cankuzo). Ethical Approval (Decision CNE/42/2022) was cleared by the National Ethics Committee. Names of participants and other personal identifiers were not included in the data collection tool. Before blood drawing, participants were explained the study objectves and they were requested to give verbal or signed individual consent forms. For the participants under 18 years, in addition to the verbal or signed informed consent from the parent or guardian, an assent was given to the adolescent to be signed as well.

## RESULTS

In this study, socio-demographic factors and risk factors for the transmission of HBV infection are listed results. A total of 629 participants took part in the study, the proportion of viral hepatitis B found in our study was 8.43%. The majority of the participants were female (57.71%). The mean age of the participants for HBV screening was 37.84 (SD=13.9) years and the mean age of the negative viral hepatitis B individuals was 40.95 (SD=15.23) years and the participants were predominantly in the age range of above 50 years (31.11%) and the age group of 18–30 (26.69%). The predominant participants were from Rumonge province city (38.31 %), had a primary education level (36.25), were married (70.06 %), and the farmers (Table 1).

**Table 1:** Distribution of Sociodemographic Characteristics (N=629)

Variables	Frequency (%)
Sex	
Male	266 (42.29)
Female	363 (57.71)
Age (years) - mean (SD)	40.69 (SD=15.14)
Age group (years)	
15-17	22 (3.50)
18-30	166 (26.39)
31-40	114 (18.12)
41-50	125 (19.87)
>50	202 (31.11)
Residence	
Gitega	210 (33.39)
Rumonge	241 (38.31)
Cankuzo	178 (28.30)
Level of education	
University	47 (7.50)
Secondary	198 (31.58)
Primary	228 (36.25)
No Formal Education	156 (24.80)
Marital status	
Single	100 (15.92)
Married	440 (70.06)
Divorced	32 (5.10)
Others	56 (8.92)
Occupation	
Famer	379 (60.25)
Government employee	200 (30.80)
Healthcare workers	14 (2.23)
NGO employee	13 (2.07)
Others	23 (3.66)
Viral Hepatitis B Infection	
Yes	53 (8.43)
No	576 (91.57)

By measuring the associations between two variables at a time, HBV infection was statistically significantly associated with age (*P=.027*), city of residence (*P=.014*), sharing sharp materials (*P=.029*) and history of vaccination against HBV (*P<.001*) (Table 2).

**Table 2:** Characteristics of Participants Stratified by Viral Hepatitis B Status (N=629)

Variables	Viral Hepatitis B Infection			
	Frequency (%)	Yes (%)	No (%)	*P Value*
Sex				.29
Male	266 (42.29)	26 (9.78)	240 (90.22)	
Female	363 (57.71)	27 (7.44)	336 (92.56)	
Age range (years)				.03
15-17	22 (3.50)	1 (4.55)	21 (95.44)	
18-30	166 (26.39)	21 (12.65)	145 (87.35)	
31-40	114 (18.120	5 (4.39)	109 (95.61)	
41-50	125 (19.87)	15 (12)	110 (88)	
>50	202 (31.11)	11 (5.45)	191 (94.55)	
City of residence				.01
Gitega	210 (33,39)	15 (7.14)	195 (92.86)	
Rumonge	241 (38.31)	14 (5.81)2	27 (94.19)	
Cankuzo	178 (28.30)	24 (13.48)	154 (86.52)	
Level of education				.18
University	47 (7.50)	4 (8.51)	43 (91.49)	
Secondary	198 (31.58)	23 (11.62)	175 (88.38)	
Primary	228 (36.25)	18 (7.89)	210 (92.11)	
No formal education	156 (24.80)	8 (5.13)	148 (94.87)	
Marital status				.06
Single	100 (15.92)	15 (15)	85 (85)	
Married	440 (70.06)	34 (7.73)	406 (92.27)	
Divorced	32 (5.10	2 (6.25)	30 (93.75)	
Others	56 (8.92)	2 (3.57)	54 (96.43)	
Occupation				.10
Farmer	379 (60.25)	27 (7.12)	352 (92.88)	
Government employee	200 (30.80)	20 (10)	180 (90)	
Healthcare workers	14 (2.23)	0 (0)	14 (100)	
NGO employee	13 (2.07)	1 (7.69)	12 (92.31)	
Others	23 (3.66)	5 (21.74)	18 (78.26)	
History of HBV vaccination				<.001
Being vaccinated	174 (27.66)	0 (0)	174 (100)	
Not vaccinated	455 (72.34)	53 (11.65)	402 (88.35)	
HIV status				.06
Positive	35 (5.56)	6 (17.14)	29 (82.86)	
Negative	594 (94.44)	47 (7.91)	547 (92.09)	
History of surgical operation	460 (73.13)	42 (9.13)	418 (90.87)	.29
History of blood transfusion	45 (7.15)	3 (6.67)	42 (93.33)	.24
History of family contact with HBV				.93
yes	116 (18.44)	10 (8.62)	106 (91.38)	
No	513 (81,56)	43 (8,38)	470 (91,62)	.59
History of multiple sexual exposure				
Multiple partner	50 (7.95)	6 (12)	44 (88)	
Polygamous	7 (1.H)	1 (14.29)	6 (85.71)	
Polyandry	18 (2.86)	1 (5.56)	17 (94.44)	
One partner	405 (64.39)	35 (8.64)	370 (91.29)	
None	149 (23.69)	10 (6.71)	139 (93.29)	
Exposure to a traditional operation practice				.68
Ear piercing	73 (11.61)	8 (10.96)	65 (89.04)	
Nose piercing	12 (1.91)	1 (8.33)	11 (91.67)	
Tattoo	17 (2.70)	0 (0)	17 (100)	
Ear and nose piercing	7 (1.H)	0 (0)	7 (100)	
None	520 (82.67)	44 (8.46)	476 (91.54)	
History of sharing sharp materials	339 (53.90)	21 (6.19)	318 (93.81)	.03

The odds of HBV infection were much higher and statistically significant with being resident of Cankuzo (P=0.014), and having a history of sharing sharp materials (*P=.03*) (Table 3). There were no statistically significant association of HBV infection with age, sex, level of education, marital status, occupation, history of surgical operation, history of blood transfusion, history of family contact with HBV, sexual multiple partners and traditional practices (Table 3).

**Table 3:** Univariate and Multivariate Analysis (N=629)

	Viral Hepatitis B Infection					
	Univariate Analysis			Multivariable Analysis Final Model		
	Crude OR	CI 95%	*P*	Adjusted OR	CI 95%	*P*
Sex						
Male	1					
Female	0.74	[0.4–1.3]	.29			
Age mean (±ET)	0.98	[0.9–1.0	.15			
Age groups (years)						
15-17	1					
18-30	3.04	[0.3–23.8]	.28			
31-40	0.96	[0.1–8.6]	.90			
41-50	2.8	[0.3–22.8]	.30			
>50	1.2	[0.1–9.8]	.80			
Residence						
Gitega	1			1		
Rumonge	0.8	[0.3–1.7]	.50	0.9	[0.4–1.9]	.80
Cankuzo	2	[1–3.9]	.04	2	[1–4.]	.04
Level of education						
University	1					
Secondary	1.4	[0.4–4.2]	.50			
Primary	0.9	[02–2.8]	.80			
Illiteracy	0.5	[0∙l–2]	.30			
Marital status	2.4	[0.9–6]	.06			
Single	1					
Married	0.4	[0.2–0.9]	.02			
Divorced	0.3	[0.08–1.7]	.20			
Others	0.2	[0.04–0.9]	.04			
Occupation						
Farmer	1					
Government employee	1.4	[0.4–2.6]	.20			
Healthcare workers	NA					
NGO employee	1	[0.1–8.6]	.90			
Others	3.6	[1.4–10.5]	.01			
Being vaccinated	1	[0.5–1.8]	.90			
HIV status						
Negative	1					
Positive	2.4	[0.9–6]	.06			
History of surgical operation						
No	1					
Yes	2.4	[0.9–6]	.06			
History of blood transfusion	1.3	[0.3–4.3]	.60			
History of sexual multiple exposure						
Multiple partner	1					
Polygamous	1.2	[0.12–11.9]	.80			
Polyandry	0.4	[0.04–3.8]	.40			
One partner	0.6	[0.2–1.7]	.40			
None	0.5	[0.18–1.5]	.20			
Exposure to a traditional operation practices						
Ear piercing	1					
Nose piercing	0.7	[0.08–6.5]	.70			
Tattoo	NA					
Ear and nose piercing	0.5	[0.3–1.6]	.40			
None	NA					
History of sharing sharps materials						
No	1					
Yes	1.8	[1.05–3.3]	.03	1.8	[1–33]	.03

## DISCUSSION

The study sought to determine the prevalence of HBV and associated factors in the population attending HBV screening programme. The proportion of those infected with HBV was high at 8.43% and the associated factors were being resident in Cankuzo and sharing sharp materials. In Burundi, data on the current prevalence of the virus of hepatitis B (VHB) in Burundian general population remains fragmentary; and according to a national survey, the prevalence of HBV at country level was estimated at 4.6% in 2002,^13^ and at 3.33% in blood donors according to Nahimana Doctoral Thesis.^14^ In the neighbouring countries (Tanzania, Rwanda, and DRC), the prevalence of HBV infection was found to be moderate as it ranges between 2 and 5%.^10–12^ The proportion of HBV infection shows a high endemicity in these three selected cities of Burundi. Similar studies showed a highly endemic hepatitis B virus infection at the community level on the African continent^20,21^. The high proportion of HBV infection could be linked to low vaccination rates in adults and limited interventions on diagnosis, prevention and treatment in the general population of the three selected cities of Burundi.

The mean age of the viral hepatitis B subjects was 37.84 (SD=13.9) years and the majority were aged above 50 years (31.11%) and in the age range of 18–30 years (26.6%). In a different study conducted in Rwanda, among the general population during an HBV screening campaign, 3.9% were HBsAg positive. The highest proportion (4.2%) was found in the 35–44-year-old group, but the difference from other groups was not significant^7^. Our findings indicate that HBV infection (8.43%) is highly endemic in this Burundian study according to World Health Organization criteria, as the prevalence could range between 2% and 8%.

The frequency of HBV infection was high in Cankuzo city (13.48 %) as presented (Table 1). The Cankuzo city has many returning refugees and other migrants from bordering countries and provinces. This population could have a higher risk of HBV acquisition due to unhealthy living conditions, low vaccination rates in adults, or possibly multicultural behaviours including sharing sharp materials. Advanced testing at the molecular level including PCR and sequencing analyses should be done to gain more understanding of the high prevalence of HBV infection and risk factors for its transmission in Cankuzo city.

The factors associated with HBsAg positivity in this study included city of residence and sharing sharps materials while the vaccination against HBV could be found as a protection factor but was not significantly associated with HBV (OR=1, CI 95%=[0.5–1.8], *P*=0.9). The same associated factors were found in other studies in Ivory Coast and Burkina Faso^22,23^. Different studies conducted in different countries identified other factors to be associated with HBV infection such as history of blood transfusion and surgery, history of vaccination uptake;^24^ and history of contact and dental procedures.^25^ Different studies found no significant association between the socio-demographic characteristics of the participants and HBV infection.^4^ In Benin, the HBV infection was found to be associated with some socio-demographic characteristics (age, sex, marital status, education level and occupation).^21^ Given the various ways for HBV infection to occur within any general population, monitoring continuous exposure and control of hepatitis B with associated risk factors is one of the highest priorities in order to mitigate its transmission in Burundian general population.

In Cameroon, being male participants, and having a history of traditional operation and scarification were associated with the presence of HBsAg in adults.^26^ In Uganda, the key influence of HBV prevalence included education level, presence of HIV infection, and number of lifetime sexual partners.^27^ These differences with our study could be explained by different methods used and diverse behaviours among people in different countries and regions. The study did not represent the whole country or provinces but has provided the proportion of Hepatitis B Virus and the magnitude of risk factors for its transmission among Burundian population attending the HBV screening campaign in the three selected cities of Burundi. This study suggests continuous screening in all other cities, vaccination in adults together with continuous medical education all over the country, particularly in high-risk provinces and regions.

## CONCLUSION

The frequency of HBV was significantly associated with residence in Cankuzo and sharing sharp materials. The HBV infection is highly endemic in the Burundian population aged 15 years and above in these three cities of Burundi. Therefore, there is a need for more emphasis on HBV screening in the general population, vaccination of adults, other preventive measures and treatment of those already infected patients. Furthermore, advanced testing at the molecular level including PCR and sequencing techniques should be done to gain more understanding of the high prevalence of HBV infection and its transmission in Cankuzo city. A population-based prevalence study also be done to provide more understanding of the current situation of HBV infection including the impact of HBV vaccination policy and existing screening strategies to further mitigate viral hepatitis B transmission in the general population.

### Study limitations

This study provides factors affecting HBV transmission in three provincial cities. It does not represent the entire Burundian population and could not be generalised. In addition, due its cross-sectional nature, the study does not reflect the discrepancies in the frequency of HBV infection in the whole year. Furthermore, self-reporting of some clinical conditions may have been affected by social desirability bias, recall bias, as well as participation bias. Nevertheless, the use of antigenic tests overcame to a large extent the self-reported biases; and the study has revealed insightful information that can be used for intervention planning and further research.
